# Reliability and validity of the Chinese version of the sunlight exposure questionnaire

**DOI:** 10.3389/fpubh.2024.1281301

**Published:** 2024-03-14

**Authors:** Xiaoxia Wang, Qin Wang, Zhe Li, Mengjie Chen, Maoting Guo, Laixi Kong, Liyuan Chen, Xiaolong Li, Junjun Li, Qieyan Cao, Zhenhua Luo, Zhenzhen Xiong, Dan Zhao

**Affiliations:** ^1^School of Nursing, Chengdu Medical College, Chengdu, China; ^2^School of Health and Medicine, Polus International College, Chengdu, China; ^3^Mental Health Center, West China Hospital, Sichuan University, Chengdu, China; ^4^Sichuan Clinical Medical Research Center for Mental Disorders, Chengdu, China; ^5^Xindu Hospital of Traditional Chinese Medicine •The First Affiliated Hospital of Traditional Chinese Medicine, Chengdu Medical College, Chengdu, China; ^6^Second Affiliated Hospital of Chengdu Medical College, Chengdu, China; ^7^Nursing Key Laboratory of Sichuan Province, Chengdu, China

**Keywords:** sunlight, vitamin D, circadian rhythms, health, reliability, validity

## Abstract

**Objective:**

This study aimed to translate and validate the reliability and validity of the Chinese version of the Philippines Sunlight Exposure Questionnaire.

**Methods:**

A total of 392 Chinese individuals aged at least 18 years, residing in various cities in Sichuan province for at least 1 year, were recruited. The reliability of the Chinese version of the questionnaire was measured through internal consistency, split-half reliability, and retest reliability, while validity was determined using the content validity index and the structure validity index.

**Results:**

The Chinese version of the Sunlight Exposure Questionnaire, which includes 19 items covering 5 factors, demonstrated McDonald’s omega coefficient of 0.788. The split-half reliability of the questionnaire was 0.823, and the retest reliability was 0.940. The content validity index (S-CVI) was 0.952. The five-factor structure, supported by eigenvalues, explained 66.2% of the total variance. Confirmatory factor analysis indicated favorable model fit.

**Results:**

The chi-square value degrees of freedom ratio (χ^2^/df) = 1.852, the goodness-of-fit index (GFI) = 0.938, the normed fit index (NFI) = 0.922, the incremental fit index (IFI) = 0.962, the comparative fit index (CFI) = 0.962, the Tucker–Lewis index (TLI) = 0.952, and root mean square error of approximation (RMSEA) = 0.047. The indicators of the fit of the model were within reasonable bounds.

**Conclusion:**

The Chinese version of the Sunlight Exposure Questionnaire shows validity and good reliability for assessing sun exposure among adults in a Chinese cultural context.

## Introduction

Sunlight is important for human health and helps regulate physiology, psychology, and behavior (1). For example, sunlight influences melatonin secretion, which in turn affects circadian pacing by the supraoptic nucleus in the brain, such that lack of natural daylight can disturb sleep–wake rhythms (2, 3). Through its effects on circadian rhythms, sunlight can influence the neuroendocrine system, cognitive function, and mood (4). Moreover, a large number of epidemiological studies have substantiated the correlation between sunlight exposure levels and a diminished risk of all-cause mortality (5), as well as various related diseases such as myopia (6), asthma (7), type 1 diabetes (8), autism (9), colorectal cancer (10), and Alzheimer’s disease (11). Additionally, a potential mechanism underlying the connection between sunlight and public health is the conversion of 7-dehydrocholesterol in the human skin into vitamin D. This process is essential for the vitamin D synthesis of the body (12, 13), a deficiency of which can contribute to osteoporosis (14), multiple sclerosis (15), diabetes mellitus (16, 17), cardiovascular diseases (18), sleep disorders (19, 20), obesity (21), depression (22), and anxiety and cognitive impairment (23, 24).

Accurate assessment of sunlight exposure in the population can help to predict the risk of related diseases so that healthcare providers can intervene in advance to minimize or even prevent problems related to inadequate or excessive exposure. Such assessments may be particularly important in the wake of the coronavirus pandemic, when many people spent much more time indoors during the daytime, increasing the risk of inadequate daily sunlight exposure. This deficiency is notably associated with diminished sleep quality (25). Similarly, such assessments may be particularly important for populations living in parts of the world that receive below-average sunlight.

Photometers and ultraviolet (UV) dosimeters can accurately measure sunlight exposure, but they are not accessible in many environments and they cannot be distributed in large numbers to analyze samples that are large or geographically dispersed. A more cost-effective measurement tool is the Sunlight Exposure Questionnaire, several of which have been developed for different linguistic and cultural contexts, including the Pakistan Sunlight Exposure Measurement Questionnaire in English and Urdu (26), the Harvard Light Exposure Assessment (27) in English, and the Philippines Sunlight Exposure Questionnaire (28) in English and Filipino. The sunlight exposure estimated from these questionnaires correlates with vitamin D levels in serum (29), supporting their reliability.

Several questionnaires to assess sunlight exposure have been developed in Chinese (30, 31). Notably, one such assessment tool, crafted by Hong Kong scholars Shenghui Wu et al. (30), stands out for its commendable questionnaire reliability and validity. This instrument aims to evaluate lifetime sunlight exposure within the Chinese population and comprises 62 items. However, the considerable time investment required for participants, particularly the Older Adult, diminishes its generalizability and applicability in real-world studies. In China, numerous studies (22, 32) have utilized the variable “sunlight exposure time” as an indicator of light exposure. Conversely, fewer studies have employed the mature Sunlight Exposure Questionnaire to assess the degree of sunlight exposure among study participants. This discrepancy may introduce inaccuracies in the assessment of individual sunlight exposure, given that various factors, such as the quantity and duration of sunlight exposure, weather conditions, outdoor activities, and sun protection measures, collectively influence individual sunlight exposure. However, they were custom-designed and have yet to be validated on large populations or other ethnic groups. We are unaware of an internationally validated questionnaire in Chinese for assessing sunlight exposure (26), which makes international comparisons difficult.

Compared to internationally available sunlight exposure questionnaires primarily designed for non-Asians, the Philippines Sunlight Exposure Questionnaire stands out, developed by Marc Gregory et al. (28) in 2018. With a more streamlined total of 25 items, this questionnaire offers items that are easy to comprehend, rendering it applicable to a broader demographic. Notably, it incorporates inquiries concerning individuals’ perceived risks and benefits associated with sun exposure, a pivotal factor in assessing an individual’s sun exposure. Furthermore, this questionnaire has undergone rigorous testing, affirming its robust reliability and validity. Given the increasing focus on the health effects of light in contemporary research, and considering the ease of administration of the questionnaire, it proves highly applicable as a research tool for assessing sunlight exposure in large cross-sectional studies. Therefore, the objective of this study was to translate the Philippines Sunlight Exposure Questionnaire (28) into Chinese and validate its reliability and validity. We validated the questionnaire in a sample of 392 adults, providing a rigorous tool for testing hypotheses about associations between sunlight exposure and human health.

## Methods

### Study design

The study is a cross-sectional study. We utilized convenience sampling to recruit a total of 392 adults from various cities in Sichuan, China, in August 2022, employing Questionnaire Star as an online data collection platform in China. The sampled cities encompassed Chengdu, Mianyang, and other urban areas within Sichuan. The validation procedure was approved by the Ethics Committee of Chengdu Medical College (2022 No. 33). The URL link to the questionnaire was posted on social media, and participants who met the following inclusion criteria were invited to click on the link and forward it to others: (1) Participants had to be at least 18 years old, (2) they had to have been living at their current address for at least 1 year, and (3) they had to be able to read and understand the text. Participants were excluded if they reported being pregnant, currently having skin disease, or being in an immunocompromised state. Participants were recruited from cities all over Sichuan province.

The minimal sample size to test the validity and reliability of the 25-item questionnaire was defined as 250 because surveying at least 10 times as many people as items can provide an adequate and stable assessment of validity (33). We decided to administer the survey to 300 people to account for losses if 20% of invalid questionnaires had to be excluded. Before completing the questionnaire, participants were provided with a detailed explanation of the purpose and significance of the study, and they were assured that their responses would be kept confidential and used only for research purposes. After providing consent, participants were able to access the questionnaire.

After submitting the questionnaires, participants were excluded if subsequent analysis suggested that they had not understood the questions, such as if they did not respond to any of the questions or they gave the same response to all items. In addition, questionnaires were excluded if at least three items were unanswered or if the participant took only 1–5 min to complete the questionnaire. If one individual submitted multiple questionnaires from different cell phones or IP addresses, only one of the questionnaires was retained.

In accordance with the recommendation of a retest reliability sample size ranging from 10 to 20% of the total sample size (34), 2 weeks after the initial survey administration, 80 respondents were invited to repeat the survey in order to assess test–retest reliability.

### Development of the Chinese version of the sunlight exposure questionnaire

We started with the Philippines Sunlight Exposure Questionnaire (28), which contains 25 items covering three factors: (1) intensity of sunlight exposure, items 1–7; (2) factors affecting sunlight exposure, items 8–19; and (3) sunlight protection measures, items 20–25. Respondents answer each item on a 4-point Likert scale comprising “never” (1 point), “sometimes” (2 points), “often” (3 points), and “always” (4 points). Scores for items within each factor are averaged to obtain the factor score, and the three-factor scores are averaged to yield an overall score. Overall scores of 1.0–2.0 indicate low sunlight exposure; >2.0–3.0, moderate exposure; and > 3.0–4.0, high exposure. Cronbach’s α coefficient in the original validation study was 0.80 (28).

### Translation process

With the developers’ permission, the Philippines Sunlight Exposure Questionnaire was initially translated into Chinese by two native Chinese speakers. One of the translators, a nurse with a master’s degree, and the other holding a master’s degree in a non-medical discipline, worked independently. Discrepancies in their translations were thoroughly discussed by the research team and the two translators, with resolutions achieved through consensus. Subsequently, the questionnaire underwent counter-translation into English by two professional English teachers. The translated versions were scrutinized, and modifications were made through group discussions. Furthermore, to enhance cultural appropriateness and maximize content validity, five nursing experts were involved in the cultural adaptation of the translated questionnaire. This expert panel, comprising two professors and three associate professors, four of whom held master’s degrees and one a doctorate, possessed an average of 19.6 ± 5.04 years of professional experience. Finally, to ensure semantic clarity and appropriateness, a preliminary survey was conducted with 20 adults using the translated questionnaire. Participants were asked to complete the questionnaire and provide feedback on its difficulty. Adjustments were made based on their comments, culminating in the final version of the Chinese adaptation of the Philippines Sunlight Exposure Questionnaire.

## Statistical analysis

Data were imported into Microsoft Excel 2010 and analyzed statistically using SPSS 26.0 (IBM, Chicago, IL, United States), AMOS 26.0 (IBM, Chicago, IL, United States), and Jamovi 2.3.28. Item analysis, validity, and reliability of the questionnaire were assessed. Results associated with *p* < 0.05 were considered statistically significant.

### Item analysis

Item analysis means to test the quality of each item, whose purpose is to test the suitability or reliability of instruments and individual items. The results can be used as the basis for the screening or modification of individual items. The analysis of items involved the utilization of the critical ratio (CR) and correlation analysis between questionnaire items and total scores. The CR was determined by ranking the total scores obtained from the questionnaire from highest to lowest. Subsequently, the total scores of the top 27% were compared with those of the bottom 27% using an independent-samples *t*-test (34). Items were removed if their CR was not statistically significant or if the correlation coefficient between the score on the item and the overall score was below 0.300 (35).

### Reliability analysis

The reliability of the questionnaire was assessed in terms of internal consistency, test–retest reliability, and split-half reliability as described (36). Internal consistency refers to the homogeneity among items and internal correlation among tools. This is evaluated through the utilization of Cronbach’s α coefficient and McDonald’s omega. Both McDonald’s omega and Cronbach’s α coefficient are employed as metrics for gauging the reliability of the scale. A score equal to or exceeding 0.7 is deemed acceptable (37, 38). Higher scores indicate better internal consistency. Test–retest reliability was expressed by calculating the Pearson correlation coefficient between the total score and each factor score to indicate the temporal stability of the questionnaire, and a correlation coefficient greater than or equal to 0.50 indicates acceptable reliability. Split-half reliability evaluates the internal consistency of the questionnaire by comparing the results of both halves of all items. A coefficient greater than 0.70 is considered satisfactory.

### Validity analysis

Validity refers to the accuracy of the scale and is assessed by convergent validity and discriminant validity. The validity of the questionnaire was assessed in terms of content validity and structural validity. The content validity index is calculated based on the values obtained from expert opinions. It was assessed in terms of the content validity index (I-CVI) and the average content validity index (S-CVI) at the item level (39). A 4-point scale was used to assess the content validity of each item, ranging from “not relevant” (1 point) to “very relevant” (4 points). I-CVI means that each item appropriately reflects the extent of the concept to be measured, and S-CVI indicates the mean value of I-CVI of all items. I-CVI ≥0.78 and S-CVI ≥0.90 are considered acceptable (39).

The construct validity was assessed using factor analysis including exploratory factor analysis (EFA) and confirmatory factor analysis (CFA). The Kaiser–Meyer–Olkin (KMO) test and Bartlett’s spherical test (χ^2^) were used to examine the suitability for factor analysis. A KMO value ≥0.80 and a significant Bartlett’s chi-square (*p* < 0.05) indicated the appropriateness of factor analysis (40). For EFA, principal component analysis and varimax rotation were used to extract the common factors (eigenvalues >1) of the questionnaire items. A factor was deleted if a load of the item on a factor was below 0.40 or load differed less than 0.05 from the load of other factors. Indicators of good construct validity included item factor loadings >0.40 and cumulative variance contributions >60%. For CFA, AMOS 26.0 (IBM, Chicago, IL, United States) was utilized to analyze the applicability of model fit indices. The model fit indices assessed in this study included the chi-square value degrees of freedom ratio (χ^2^/df), goodness-of-fit index (GFI), comparative fit index (CFI), incremental fit index (IFI), normed fit index (NFI), and Tucker–Lewis index (TLI). Additionally, the root mean square error of approximation (RMSEA) was considered. The criteria for a good fit were χ^2^/df < 3.000, and GFI, CFI, IFI, NFI, and TLI values were above 0.90, with RMSEA values <0.08.

## Results

### Demographic characteristics

Of the 424 questionnaires received, 32 were excluded because 22 were incomplete and 10 contained the same response to all items. The final analysis contained 392 questionnaires, primarily from women (303, 77.3%). Across all participants, the average age was 30.3 ± 9.1 years, and 236 (60.2%) had completed secondary school (Table 1). The sample represented a diverse range of occupations, such as students (90, 23%), healthcare workers (80, 20.4%), freelancers (64, 16.3%), farmers (51, 13%), and government workers (10, 2.6%). Most respondents indicated that they worked indoors (267, 68.1%) or a combination of indoors and outdoors (104, 26.5%). All respondents came from Sichuan province, with nearly half (162, 41.3%) living in Chengdu and 116 (29.6%) living in Mianyang.

**Table 1:** Characteristics of participants in the validation study (*N* = 392).

Characteristics	*n* (%)
**Sex**	
Male	89 (22.7)
Female	303 (77.3)
**Education level**	
Undergraduate and above	182 (46.4)
College	54 (13.8)
Secondary/high school	70 (17.9)
Junior high school	75 (19.1)
Elementary school and below	11 (2.8)
**Occupation**	
Government worker	10 (2.6)
Healthcare worker	80 (20.4)
Other type of worker	42 (10.7)
Teacher	10 (2.6)
Freelancer	64 (16.3)
Student	90 (23)
Farmer	51 (13.0)
Other	45 (11.5)
**Type of work**	
Day shift	280 (71.4)
Night shift	1 (0.3)
Both shifts	111 (28.3)
**Workplace**	
Outdoor	21 (5.4)
Indoor	267 (68.1)
Both	104 (26.5)
**Place of residence**	
Chengdu	162 (41.3)
Mianyang	116 (29.6)
Other city	114 (29.1)

### Item analysis

The results of this study showed that the CR, which ranged from −2.650 to −12.369, was significant for all items except items 24–25, and the absolute value of CR of item 1 was less than 3 (Table 2). Additionally, we also examined the correlations of scores on each item with the overall score. Items 1, 2, 7, 23, 24, and 25 had correlation coefficients <0.300 (Table 2), with *p* > 0.05 for the correlation coefficients of items 24 and 25 (Table 2). As relevant literature (35), the deletion criteria included items with an absolute value of CR < 3 and p > 0.05 as well as items with a correlation coefficient < 0.3 and *p* > 0.05. Such items are considered insufficient in discriminating between high and low scores, displaying less homogeneity with other items and inadequate discriminatory capacity, leading to their proposed deletion. Consequently, items 1, 2, 7, 23, 24, and 25 were suggested for removal due to not meeting the retention criteria from a statistical perspective. Despite the statistical recommendations, caution is advised when deleting items, as the process involves a combination of statistical knowledge and professional background knowledge. Following expert group discussions, it was agreed that the content of item 7 (“What time of day are you usually exposed to the sun?”) was closely related to individuals’ degree of sunlight exposure. Therefore, based on a comprehensive evaluation of statistical results, item semantics, and professional significance, the analysis led to the retention of item 7 and the deletion of items 1, 2, 23, 24, and 25. The 20 retained items demonstrated better differentiation and homogeneity, aligning with the anticipated discrimination and homogeneity outcomes reflected in the survey results. Therefore, we decided to evaluate whether the remaining factors should be deleted based on the analyses of the exploratory and validation factors (see below) as well as theoretical considerations.

**Table 2:** Critical ratios for each item and their correlation coefficients with overall score on the Chinese version of the sunlight exposure questionnaire.

Item no.	Critical ratio (*t*-value*)	Correlation coefficient (*r*)
1	−2.650	0.151
2	−3.344	0.243
3	−9.277	0.498
4	−8.775	0.498
5	−9.099	0.486
6	−7.429	0.453
7	−4.100	0.265
8	−10.769	0.528
9	−8.363	0.452
10	−6.969	0.364
11	−6.804	0.418
12	−7.387	0.420
13	−8.140	0.426
14	−5.985	0.355
15	−8.210	0.429
16	−8.225	0.438
17	−9.321	0.455
18	−12.369	0.496
19	−10.401	0.465
20	−5.260	0.330
21	−6.415	0.344
22	−6.038	0.348
23	−4.971	0.290
24	0.381 (*p* > 0.05)	0.017 (*p* > 0.05)
25	−0.060 (*p* > 0.05)	0.038 (*p* > 0.05)

*Refers to the *t*-value for the comparison between the score on the given item among respondents whose overall questionnaire scores fell among the top 27% scores and the score among respondents with overall scores among the bottom 27%.

### Reliability analysis

#### Internal consistency

For the Chinese version of the Sunlight Exposure Questionnaire, McDonald’s omega coefficient was 0.788. Cronbach’s α coefficient for each factor ranged from 0.668 to 0.861, Cronbach’s α coefficient was 0.779, and deleting each item one by one from the analysis did not increase Cronbach’s α coefficient, indicating good internal consistency of the Chinese version of the Sunlight Exposure Questionnaire.

#### Test–retest reliability

Test–retest reliability was 0.940 based on total score correlation analysis.

#### Split-half reliability

For the 19-item version, split-half reliability was 0.823 for the total questionnaire, and the reliability ranged from 0.669 to 0.838 across the different factors.

### Validity analysis

#### Content validity

For the expert panel, I-CVI ranged from 0.800 to 1.000, and S-CVI was 0.952 for the overall questionnaire.

#### Construct validity

##### Exploratory factor analysis

We did not remove any of the 25 items before performing exploratory factor analysis. We confirmed that such analysis could be performed because the KMO value was 0.811, greater than the cut-off of 0.800 (40), and χ^2^ = 3,748 (*p* < 0.001) in Bartlett’s spherical test (40). We analyzed the factor structure in terms of principal components and maximum variance with orthogonal rotation. Items with factor loadings <0.40 were removed, while items that loaded >0.40 onto more than one factor were assigned to the factor most conceptually related to them. After exploratory factor analysis with variance maximization and orthogonal rotation, item 10 (with a factor loading <0.4) was removed (34). Five common factors with initial eigenvalues >1 were extracted according to the Kaiser criterion (41), with a cumulative variance contribution of 66.2%. The factor loadings of each item ranged from 0.644 to 0.854, and there were no double loadings.

All items were retained in the validated factor analysis, except items 1, 2, 10, 23, 24, and 25. A total of five factors were defined: sunlight exposure intensity, items 3–7; outdoor exposure to sunlight, 8–9; factors promoting sunlight exposure, 11–14; factors reducing sunlight exposure, 15–19; and measures that protect against sunlight, 20–22. The final Chinese version of the questionnaire included 19 items covering 5 factors. The factor “factors affecting sunlight exposure” in the original questionnaire was divided into three factors in the Chinese version (see Table 3).

**Table 3:** Factor loading on the final Chinese version of the sunlight exposure questionnaire.

Item*	Factor				
	Intensity of sunlight	Outdoor exposure to sunlight	Factors that promote sunlight exposure	Factors that reduce sunlight exposure	Measures that protect against sunlight
Q3周一至周五，您通常花多长时间晒太阳? How long do you usually spend under the sun during the week (Monday to Friday)?	**0.824**	0.184	0.118	−0.015	0.026
Q4周六或周日，您通常花多长时间晒太阳? How long do you usually spend under the sun on the weekend (Saturday and/or Sunday)?	**0.811**	0.099	0.193	−0.005	−0.027
Q5晴天，您通常花多长时间晒太阳? How long do you usually spend under the sun when the weather is sunny?	**0.854**	0.152	0.172	−0.063	−0.025
Q6多云天，您通常花多长时间晒太阳? How long do you usually spend under the sun when the weather is cloudy?	**0.791**	0.190	0.099	−0.076	0.003
Q7您每天通常在什么时间晒太阳? At what time of the day are you usually exposed to the sun?	**0.644**	−0.166	0.044	0.006	−0.217
Q8您因工作或日常生活外出而晒太阳的频率是? How often do you go out in the sun due to work or daily routine?	0.473	**0.652**	0.047	0.096	0.022
Q9您步行或者乘坐剬共交通工具去工作或日常活动外出的频率是? How often do you walk or use public transport to do the above activities?	0.167	**0.848**	0.000	0.088	0.142
Q11您在补钙的同时额外补充维生素D或复合维生素的频率是? How often do you take calcium with vitamin D supplements or multivitamins?	0.168	−0.117	**0.651**	0.107	0.115
Q12您会为了强健骨骼，促进健康而去晒太阳吗? How likely are you to be exposed to the sun to get stronger bones and better health?	0.106	0.033	**0.849**	−0.007	0.016
Q13您会为了更快乐，更有活力而去晒太阳吗? How likely are you to be exposed to the sun to get happier and livelier?	0.165	0.125	**0.776**	−0.032	0.027
Q14您会为了更好看的皮肤(小麦色)而去晒太阳吗? How likely are you to seek sun exposure to have more beautiful skin?	0.113	−0.013	**0.752**	0.131	−0.178
Q15您会因为受到家人、朋友和同事的影响而不去晒太阳吗? How likely are you to avoid sun exposure due to the influence of family, friends, and coworkers?	−0.084	0.169	0.346	**0.675**	−0.142
Q16您会因为受到电视、广播和互联网的影响而不去晒太阳吗? How likely are you to avoid sun exposure due to the influence of TV, radio, and the internet?	−0.104	0.110	0.288	**0.723**	−0.083
Q17您会因为有晒伤、皮肤癌、皮肤过敏和皮疹的可能而不去晒太阳吗? How likely are you to avoid sun exposure due to the possibility of sunburn, skin cancer, skin allergy, and rash?	0.050	−0.099	−0.028	**0.773**	0.227
Q18您会因为有中暑、高血压和头晕的可能而不去晒太阳吗? How likely are you to avoid sun exposure due to the possibility of heat stroke, hypertension, and dizziness?	0.026	0.004	−0.115	**0.764**	0.351
Q19您会因为有出汗和被晒黑的可能而不去晒太阳吗? How likely are you to avoid sun exposure due to the possibility of sweating and fear of darker skin?	−0.058	0.149	−0.175	**0.671**	0.477
Q20当您外出晒太阳时，您戴帽子的频率是? When going out in the sun, how often do you wear a hat?	0.070	−0.169	0.122	0.047	**0.748**
Q21当您外出晒太阳时，您打伞的频率是? When going out in the sun, how often do you use an umbrella?	−0.156	0.134	0.004	0.190	**0.762**
Q22当您外出晒太阳时，您在阴凉处行走的频率是? When going out in the sun, how often do you walk in the shade?	−0.112	0.245	−0.112	0.144	**0.710**
Eigenvalue	4.310	1.071	2.279	3.535	1.391
Cumulative variance contribution (%)	22.685	66.246	53.286	41.289	60.608

*Numbered according to their position in the original questionnaire. Bolded values indicate factor loadings > 0.6, reflecting significant factor indicators.

##### Confirmatory factor analysis

**Figure 1:**
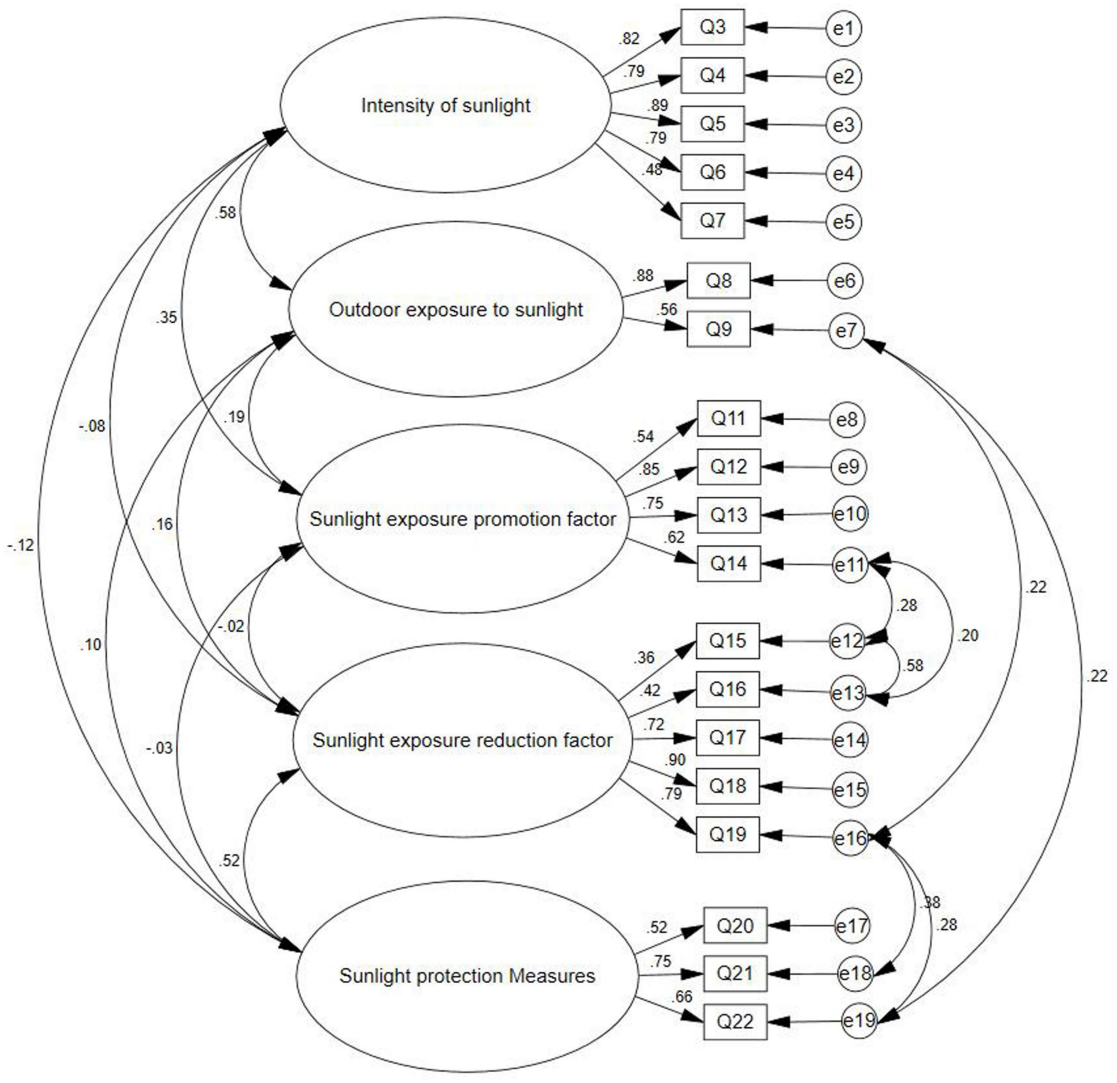
Standardized factor structure of the final Chinese version of the sunlight exposure questionnaire.

The reasonableness of the five-factor model was assessed using confirmatory factor analysis after the addition of seven residual paths (Figure 1). Construct validity based on model fit metrics showed that the model fit was good. The quality indicators of the model were within the ranges defined as satisfactory: (42) χ^2^/df = 1.852 (< 3.000), RMSEA was 0.047 (< 0.080), GFI was 0.938 (> 0.900), CFI was 0.962 (> 0.900), IFI was 0.962 (> 0.900), NFI was 0.922 (> 0.900), and TLI was 0.952 (> 0.900). All standardized path regression coefficients exceeded 0.40, ranging from 0.51 to 0.86.

## Discussion

We introduce and validate a Chinese adaptation of the Philippines Sunlight Exposure Questionnaire (28). The 19-item Chinese version encompasses 5 factors. Through our validation involving nearly 400 Chinese adults, the questionnaire demonstrates suitable convergent and discriminant construct validity, internal consistency, and test–retest reliability.

This survey is, to our knowledge, the first rigorously validated questionnaire for assessing sunlight exposure in adults. It may therefore provide a more solid basis for studies benchmarking Chinese populations against samples in other countries according to sunlight exposure, attitudes to such exposure, potential protective measures, and potential effects on health. Previously reported questionnaires in Chinese for assessing sunlight exposure (30, 31) were validated qualitatively but not quantitatively as in the present study.

Item 1 (“How do you describe your skin when it is exposed to the sun?”) and item 2 (“What part of your body is usually exposed to the sun?”) of the original questionnaire did not survive our validated factor analysis with a sample of Chinese adults. This may reflect that many Chinese know that UV exposure affects skin tone and causes sunburn, but their understanding may be vague and not based on science (43, 44). In any case, self-reported photoresponse may correlate weakly or not at all with actual photoresponse (45), so deletion of the original items 1–2 from our survey may not lead to loss of information in Chinese samples.

We also deleted item 10 from the original questionnaire (“How often do you engage in outdoor activities such as jogging, cycling, and swimming?”). This item showed little correlation with other items. This may reflect that most cities in Sichuan province have similarly warm and humid weather year-round, leading many to spend more time indoors (46). In addition, many in our sample were students, for whom the first priority may be studying, rather than exercising, even if they recognize the importance of exercise to their health (47, 48). Our deletion of item 23 (“When going out in the sun, how often do you use sunscreen containing at least SPF 30?”), item 24 (“When do you usually apply sunscreen?”), and item 25 (“Where do you usually apply sunscreen?”) from the original questionnaire may reflect the relatively low rate of use of sunscreen among Chinese adults, reflecting in part low awareness of the hazards of sunlight exposure (49, 50). Many Chinese adults may prefer to protect their skin by staying in the shade or wearing hats, rather than using sunscreen (49), which may reflect a Chinese preference for fair skin over tanned skin (51). Indeed, the original questionnaire focused on Manila (121°E, 15°N), which receives more sunlight than Sichuan province, where the major cities are Chengdu (102°54′–104°53′E, 30°05′–31°26′N) and Mianyang (103°45′–105°43′E, 30°42′–33°03′N).

The Chinese version of the Sunlight Exposure Questionnaire takes only minutes to complete, making it a straightforward and rigorous tool for surveying adults in the general population as well as in clinics and care homes. The questionnaire was employed in the following manner: First, participants were instructed to complete the questionnaire. Subsequently, participants’ sun exposure was evaluated based on the questionnaire scores. Overall scores falling within the range of 1.0–2.0 indicate low sunlight exposure, scores >2.0–3.0 indicate moderate exposure, and scores >3.0–4.0 indicate high exposure. In the contemporary landscape, there exists substantial public interest in understanding the risks and benefits associated with sun exposure. The potential utilization of this questionnaire for quantifying an individual’s exposure to sunlight opens avenues for new research opportunities. This may facilitate investigations into the relationship between sunlight exposure and health outcomes, offering insights into the advantageous aspects of sunlight exposure and contributing to the prevention or delay of the onset and progression of related diseases. The data gained with this questionnaire may help guide efforts to identify individuals at risk of inadequate sunlight exposure, which can increase the risk of morbidity (52), such as inflammatory bowel disease (53) or osteoporotic fractures (54). Measures to increase sunlight exposure of at-risk individuals can then be encouraged. Conversely, the questionnaire can identify individuals who receive abundant sunlight but may not protect themselves adequately, and who therefore may benefit from interventions that sensitize them to the dangers of excessive sunlight and educate them about appropriate protection measures. In this study, a significant proportion of participants dedicated more time indoors, primarily for study, living, and work-related reasons associated with their occupations. While this practice may mitigate excessive sunlight exposure, it raises concerns about potential insufficient sunlight exposure for these individuals. Therefore, the assessment of individual sun exposure becomes crucial. Accurate knowledge of specific sunlight exposure is imperative to implement measures aimed at enhancing public exposure to sunlight, a necessity for promoting both physical and mental health in the general population.

This study has several limitations. First, as this study was conducted during the COVID-19 epidemic, the sample was obtained through convenience sampling using an online questionnaire, resulting in a predominantly female, Sichuan-based, and urban-dwelling respondent pool, leading to a sex distribution imbalance. Future studies will have to strive to recruit more male participants and rural residents for a more comprehensive assessment of the Chinese version of the Sunlight Exposure Questionnaire across genders and regions in China. The second limitation is the absence of objective standardized measures of sunlight exposure, such as serum 25-hydroxyvitamin D (25-OHD) measurements. Skin synthesis via UV exposure serves as the primary source of vitamin D, and researchers have employed vitamin D measurements as a clinical tool for evaluating individual sun exposure in various studies (55–57). Therefore, follow-up studies incorporating serum 25-OHD measurements could enhance the scientific accuracy of the investigations. The correlation between sunlight exposure questionnaires and serum vitamin D values could be further validated in subsequent studies.

## Conclusion

Following translation and cross-cultural adaptation, the Philippines Sunlight Exposure Questionnaire has been introduced in China, demonstrating good reliability and validity. The Chinese version of the Sunlight Exposure Questionnaire is deemed suitable for evaluating sunlight exposure in Chinese adults. Moreover, the questionnaire serves as a valuable reference for health promoters in the development of educational programs and research interventions aimed at promoting the physical and mental health of the public.

## Data availability statement

The raw data supporting the conclusions of this article will be made available by the authors, without undue reservation.

## Ethics statement

The studies involving humans were approved by Ethics Committee of Chengdu Medical College. The studies were conducted in accordance with the local legislation and institutional requirements. The participants provided their written informed consent to participate in this study.

## Author contributions

XW: Investigation, Writing – original draft. QW: Writing – original draft, Investigation. ZLi: Investigation, Writing – original draft. MC: Investigation, Writing – review & editing. MG: Investigation, Writing – review & editing. LK: Investigation, Writing – review & editing. LC: Investigation, Writing – review & editing. XL: Investigation, Writing – review & editing. JL: Investigation, Writing – review & editing. QC: Investigation, Writing – review & editing. ZLu: Project administration, Writing – review & editing. ZX: Methodology, Supervision, Writing – review & editing. DZ: Project administration, Writing – review & editing.
